# Extracranial carotid artery atherosclerotic plaque and APOE polymorphisms: a systematic review and meta-analysis

**DOI:** 10.3389/fcvm.2023.1155916

**Published:** 2023-11-13

**Authors:** Sinéad Culleton, Mary Niu, Matthew Alexander, J. Scott McNally, Chun Yuan, Dennis Parker, Hediyeh Baradaran

**Affiliations:** ^1^ Department of Radiology and Imaging Sciences, University of Utah, Salt Lake, UT, United States; ^2^ Department of Pediatrics, University of Utah, Salt Lake, UT, United States

**Keywords:** atherosclerosis risk factors, carotid artery arteriosclerosis, genetic susceptibility, lipoprotein, meta-analysis

## Abstract

**Introduction:**

Carotid atherosclerotic plaque is an important independent risk factor for stroke. Apolipoprotein E (APOE) influences cholesterol levels and certain isoforms are associated with increased carotid atherosclerosis, though the exact association between APOE and carotid plaque is uncertain. The study aimed to evaluate the association between APOE and carotid plaque.

**Methods:**

A systematic review was performed to retrieve all studies which examined the association between carotid plaque and APOE. This study was conducted in accordance with the PRISMA guidelines. Independent readers extracted the relevant data from each study including the type of imaging assessment, plaque definition, frequency of APOE E4 carrier status and type of genotyping. Meta-analyses with an assessment of study heterogeneity and publication bias were performed. Results were presented in a forest plot and summarized using a random-effects model.

**Results:**

After screening 838 studies, 17 studies were included for systematic review. A meta-analysis of 5 published studies showed a significant association between *ε*4 homozygosity and carotid plaque [odds ratio (OR), 1.53; 95% CI, 1.16, 2.02; *p *= .003]. Additionally, there was a significant association between patients possessing at least one *ε*4 allele, heterozygotes or homozygotes, and carotid plaque (OR, 1.25; 95% CI, 1.03, 1.52; *p *= .03). Lastly, there was no association between *ε*4 heterozygosity and carotid plaque (OR, 1.08; 95% CI, 0.93, 1.26; *p *= .30).

**Conclusion:**

APOE *ε*4 allele is significantly associated with extracranial carotid atherosclerotic plaque, especially for homozygous individuals.

## Introduction

Research on the apolipoprotein E gene (gene = APOE, protein = apoE), has continued to mount with sustained effort to better understand its role in neurodegenerative and vascular pathology. To date, APOE has been linked to Alzheimer's dementia, age-related cognitive decline, stroke, and cardiovascular disease (1–4). The APOE gene codes for the glycoprotein product apolipoprotein E protein (apoE) and the *ε* polymorphism located in exon 4 has been most investigated (5). The three common isoforms E2, E3, and E4 are coded for by the same gene locus differing by single amino acid changes at positions 112 and 158 of the protein sequence (6). This sequence difference leads to significant isoform functional differences (6). Isoforms are encoded by the alleles *ε*2, *ε*3, and *ε*4, together constituting six possible genotypes (7). The *ε*3 allele and *ε*3/ε3 genotype are the commonest, occurring in one-half to one-third of people in most populations (8).

The apoE proteins mediate neurodegenerative and vascular diseases through several mechanisms including altering amyloid *β* clearance, affecting cholesterol homeostasis, and increasing neuroinflammation (9). The *ε*4 allele is most associated with Alzheimer's dementia, ischemic heart disease, and increased total cholesterol levels (4, 5, 7, 10). Apo E polymorphisms have garnered much attention in the hope of better understanding the pathogenesis of atherosclerosis. Cholesterol levels are influenced by APOE through the apoE isoforms which interact differently with lipoprotein receptors to play a major role in lipid transport and metabolism (8, 11). Because elevated lipids increase the risk of coronary heart disease, APOE has been widely investigated in disorders of elevated cholesterol or lipids (7).

Studies have examined how APOE-driven hyperlipidemia modulates susceptibility to atherosclerosis. APOE is associated with increased carotid intima-media thickness (C-IMT), a marker of subclinical atherosclerosis which is independently associated with myocardial infarction and stroke (7). The influence of apoE polymorphisms on C-IMT, however, has had conflicting results (12, 13). Carotid artery plaque is a further marker of atherosclerosis and is independently associated with stroke (14, 15). There is some evidence of an association between apoE polymorphisms and increased carotid plaque formation, though the exact association between apoE polymorphisms and carotid plaque is not clear, given conflicting results from multiple studies (12, 16).

To bridge this gap in understanding, a systematic review and meta-analysis of studies on adult patients were conducted to examine the association between carotid artery plaque formation and APOE polymorphisms with the hypothesis that individuals with the *ε*4 allele will be most likely to have carotid plaque.

## Methods

The Cochrane Handbook for Systematic Reviews of Interventions (17) was consulted for methodological guidance. This systematic review and meta-analysis were conducted following the Preferred Reporting Items for Systematic Reviews and Meta-Analyses: PRISMA statement (18–20). The protocol for this systematic review and meta-analysis was not registered.

### Data searches

A sensitive search was developed for Medline, which was selected as the primary database, and subsequently adapted the subject headings and keywords for other databases (see Supplemental Materials for search methodology). The following databases were searched from inception to May 3rd, 2022: Medline (Ovid), Embase (embase.com), Cochrane Library (wiley.com) including CENTRAL (wiley.com), CINAHL Complete (Ebscohost), PsycINFO (Ebscohost), Scopus (scopus.com). The references of selected studies were checked for eligibility. Studies published in languages other than English were included if an English translation was available. Grey literature was not searched. EndNote (Clarivate Analytics) was used to manage citations and remove duplicates.

This review sought to include all available published studies on APOE polymorphisms and carotid plaque in adult humans. The eligibility criteria for studies included in this review were: (1) studies that used ultrasound (US), computed tomography angiography (CTA), or magnetic resonance angiography (MRA) of the cervical common and internal carotid arteries to assess plaque; (2) studies that performed genetic testing for the Apolipoprotein *ε* allele; and (3) studies that correlated apolipoprotein *ε* allele carrier status to carotid plaque. Studies were excluded if (1) non-human studies, (2) patients <18 years, (3) did not use imaging to evaluate carotid plaque, and (4) did not test for Apolipoprotein E. If authors had published multiple manuscripts from a single study cohort or dataset, the manuscript with the largest sample size was included to prevent duplication or overlapping population samples.

### Data extraction

All potentially eligible titles and abstracts were initially reviewed by two readers (HB a neuroradiologist, MN a pediatric cardiologist, with >10 years of experience). The full articles were obtained for all potentially relevant studies. Two independent readers (HB, and SC, neuroradiologists with >10 years of experience) screened these articles in their entirety to determine eligibility for inclusion and extracted the information and data from each study. Any disagreements and uncertainties where possible were resolved using discussion and mutual consensus. When conflicts could not be resolved between the two reviewers, a third reviewer cast the deciding vote. Data were extracted by two independent readers using pre-specified data-collection templates in Excel (Microsoft version 16.62) as detailed in the Methodological Expectations of Cochrane Intervention Reviews (18)*.* For each study, the two readers independently extracted information on the year of publication, the country in which the study was conducted, the type of study, the study population, mean age, gender distribution, cardiovascular risk factors, cardiovascular medications, method of plaque measurement, plaque definitions, and method of genotyping. If key information or data were not presented in the relevant publications, data were sought directly from the authors.

The E2, E3 and E4 genotype groups were defined as follows: E2 homozygotes (*ε*2/ε2) or heterozygous (*ε*2/ε3 and *ε*2/ε4), E3 (*ε*3/ε3), E4 similarly as (*ε*4/ε4, *ε*4/ε3). The following bias assessment criteria were used (1) risk of outcome ascertainment bias was assessed by recording whether researchers were blinded to genetic characteristics; (2) risk of confounding bias was assessed by recording whether potentially confounding vascular risk factors were collected and statistically analyzed; (3) completeness of data was assessed by noting if the selection criteria for the study's population were adequately described. The risk of bias was assessed by the consensus of two readers using Joanna Brigg's Institute critical appraisal checklist (21).

### Data analysis

Meta-analyses of each study's odds ratio were conducted with the Cochrane's Review Manager (Revman, Version 5.4, The Cochrane Collaboration 2020). Pooled odds ratios (OR) were calculated with a random-effects (DerSimonian and Laird) model (22) as this approach incorporates the heterogeneity of effects in the analysis, and forest plots were generated to display the individual odds ratios. Results from each study are expressed as OR with a 95% confidence interval. Heterogeneity was calculated using the Cochrane Q and *I*^2^ statistical heterogeneity tests. Publication bias was quantitatively assessed using Egger's regression test (23). Additionally, publication bias was assessed by visual inspection of funnel plots of the OR plotted against the presence of carotid plaque examining for asymmetry. A sensitivity analysis using the leave-out-one method was performed to assess if the pooled size effect changed after eliminating one study successively. A meta-regression helps to identify variables associated with an increased pooled heterogeneity. Given the small number of studies, we had to limit this to using one variable at a time, univariable meta-regression. When two variables were attempted, the models were unreliable due to overfitting. Even the univariable models were sometimes unreliable, reporting adjusted R2 values of 100% even when no heterogeneity was explained, but we reported them for completeness. *P*-values < .05 were considered statistically significant.

## Results

**Figure 1:**
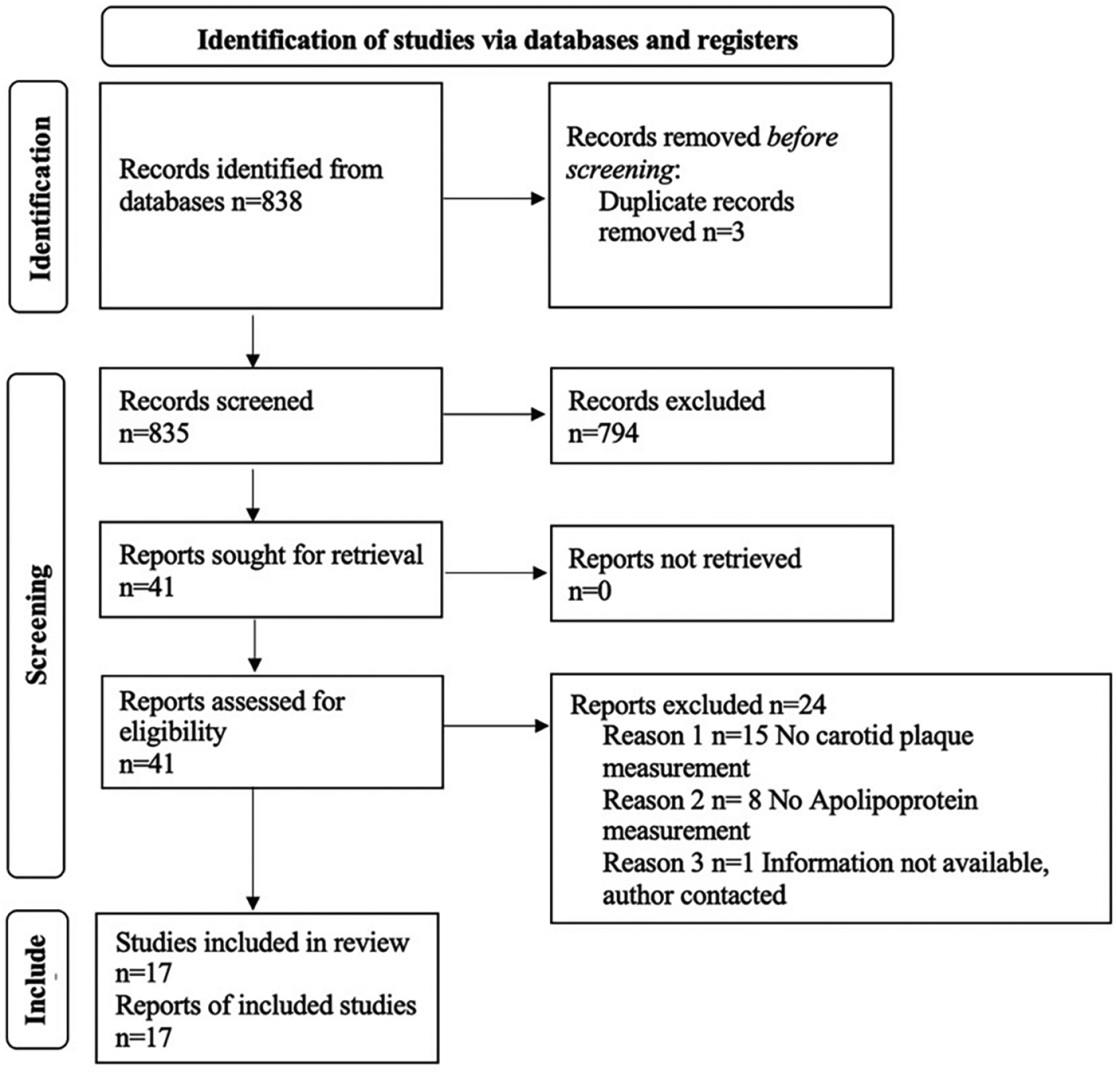
PRISMA flow diagram.

The search strategy yielded 838 records after removing duplicates (Figure 1). Forty-one full-text studies were selected as potentially eligible articles for further review. After screening and exclusions, 17 studies were included in the systematic review (Table 1). Of these 17 articles satisfying the inclusion criteria for systematic review, all were prospective cross-sectional studies.

**Table 1:** Summary of the studies included in the meta-analysis.

Study	Size *N*	Age Years	Genotyping method	Imaging modality	*ε*4 allele Frequency *N* or %	Odds ratio E4	Odds ratio ε4/ε4	Odds ratio ε3/ε4
Asakimori et al. (27)	163	54.5	PCR/RFLP	Ultrasound	32	1.62 (0.57–4.43)	–	–
Beilby et al. (12)	1,109	52.5	PCR	Ultrasound	14.7%	–	2.85 (0.49–16.57) Males, 1.15 (0.21–6.31) Females	1.79 (1.01–3.17) Males 0.63 (0.34–1.20) Females
Calmarza et al. (26)	171	68	PCR	Ultrasound	30	0.839 (0.300–2.345)	–	–
Debette et al. (5)	5,856	73.5	PCR	Ultrasound	11%	–	2.12 (1.27−3.53)	1.08 (0.93–1.25)
Djousse et al. (25)	554	56.4	PCR	Ultrasound	14%	1.1 (0.7–1.9)	–	–
Doliner et al. (13)	1,243	69	PCR	Ultrasound	14%	1.16 (0.87–1.54)	–	–
Fernandez-Miranda et al. (16)	225	60.9	PCR	Ultrasound	22%	0.5 (0.2–1.1)	–	–
Hsieh et al. (29)	479	≥40	PCR/RFLP	Ultrasound	89	–	2.0 (1.2–3.2)	–
Shin et al. (32)	19,201	63.3	PCR	Ultrasound	9%	1.08 (0.99–1.18)	1.14 (0.82–1.58)	1.08 (0.99–1.18)
Slooter et al. (33)	5,401	69.2	PCR	Ultrasound	1,529	–	1.3 (0.7–2.2)	1.09 (0.8–1.2)

### Characteristics of included studies and subjects

Demographic details and study characteristics are shown in Supplementary Table S1. In total, there were 36,245 subjects (sample size ranging from 75 to 19,201) with 15,285 (42.2%) males and 20,960 (57.8%) female subjects. Geographically, three studies were conducted in the United States (13, 24, 25), two in Spain (16, 26), one each in Japan (27), Australia (12), Poland (28), France (5), Taiwan (29), Turkey (30), Finland (31), Korea (32), the Netherlands (33), Greece (34), Italy (35), and Serbia (36). The majority were single-center studies, with three conducted at multiple sites (5, 25, 32). Six studies drew subjects from population samples (5, 12, 26, 29, 32, 33) and the remaining studies evaluated specific populations including 1 study that sampled patients with coronary disease (16), one examined subjects after carotid endarterectomy (36), one looked at patients with chronic kidney disease on hemodialysis (27), one included post-transplant individuals (30), one with patients with ischemic stroke within seven days of onset (28), two evaluated males with hypertension (24, 31), one had families with higher-than-expected rates of coronary heart disease (25), one enrolled menopausal women (34), and one examined mildly cognitively impaired individuals (35). Of the 17 studies in the systematic analysis, 10 were eligible for meta-analysis (5, 12, 13, 16, 25–27, 29, 32, 33). The seven excluded studies (24, 28, 30, 31, 34–36) were not amenable to calculations for the pooled odds ratios. The studies included in the meta-analysis are summarized in Table 1.

### Carotid imaging

Ultrasound was chosen by all studies to examine the carotid arteries (typically the common carotids, bifurcation, and proximal internal carotid arteries) for the presence of plaque. Imaging examinations were predominantly conducted using B-mode ultrasound with at least a five MHz transducer (5–13 MHz), the majority utilized a 7.5 MHz probe (12, 16 27, 29–33, 35) (Supplementary Table S2).

### Definitions of carotid plaque

The commonest definition of plaque was a protrusion into the carotid lumen, quantified as more than 50% greater than the surrounding thickness (13, 31, 35), with a cut-off ≥1 mm (5, 12, 28, 29), >1.2 mm (16, 34), or ≥1.5 mm (27, 30) maximum intima-media thickness. Visual inspection was used to estimate thickness relative to the adjacent IMT in four studies (24–26, 31), as 50% (16, 31, 35), 100% (32) or 200% (26) of the surrounding site. Two studies defined plaque as a focal widening relative to the adjacent segment (33, 36). Plaque echogenicity was specified in three studies (24, 26, 36). Six studies reported that the ultrasound operators were blinded to all clinical details (26, 27, 29–32).

### Genotyping

Genotyping was carried out on DNA extracted from blood samples using the polymerase chain reaction (PCR) method for 16 studies (5, 12, 13, 16, 24–30, 32–36). The restriction digestion enzymes included Hhal (5, 16, 24, 25, 27–29, 32, 33), Cfol (30) and Hin6I (36). PCR restriction fragment length polymorphism was used in four studies (27–29, 36). One study used an isoelectric and immunoblotting technique (31) (Supplementary Table S3). The frequency with which the *ε*4 allele was present included 9% (32), 11% (5), 12.0% (30), 12.6% (24), 14.0% (13, 25), 14.7% (12), 18.9% (31), and 22% (16).

### Meta-Analysis

**Figure 2:**
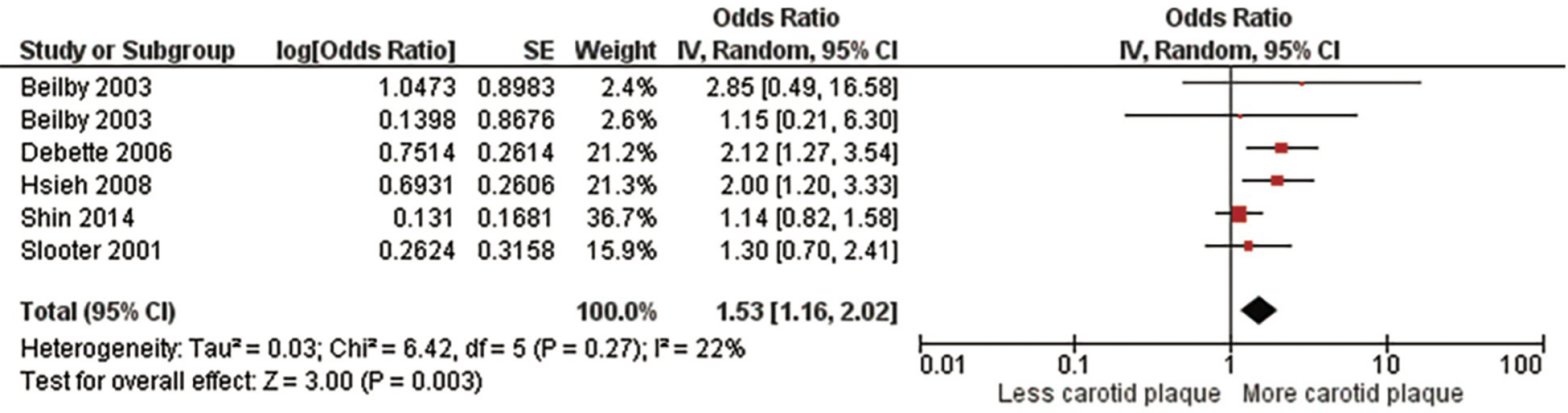
Association between ϵ4 homozygotes and carotid plaque.

Three meta-analyses were performed. The first meta-analysis examined the association between homozygotes (*ε*4/ε4) and the presence of carotid plaque (Figure 2, Supplementary Table S4, Supplementary Figure S1). For the meta-analysis evaluating the strength of the association between subjects with *ε*4 homozygosity and the presence of carotid plaque, 32,046 subjects from five studies (5, 12, 29, 32, 33) were included. There was a significant positive association between the *ε*4 homozygosity and the presence of carotid plaque with a pooled OR of 1.53 (95% CI: 1.16, 2.02), *p *= .003. There was no significant heterogeneity, Chi2, 6.42; *I*^2^, 22%; (*p *= .27). After sensitivity analysis with every instance the conclusion did not change the study's original conclusion (*p* values ranged from <.0001 to.08).

**Figure 3:**
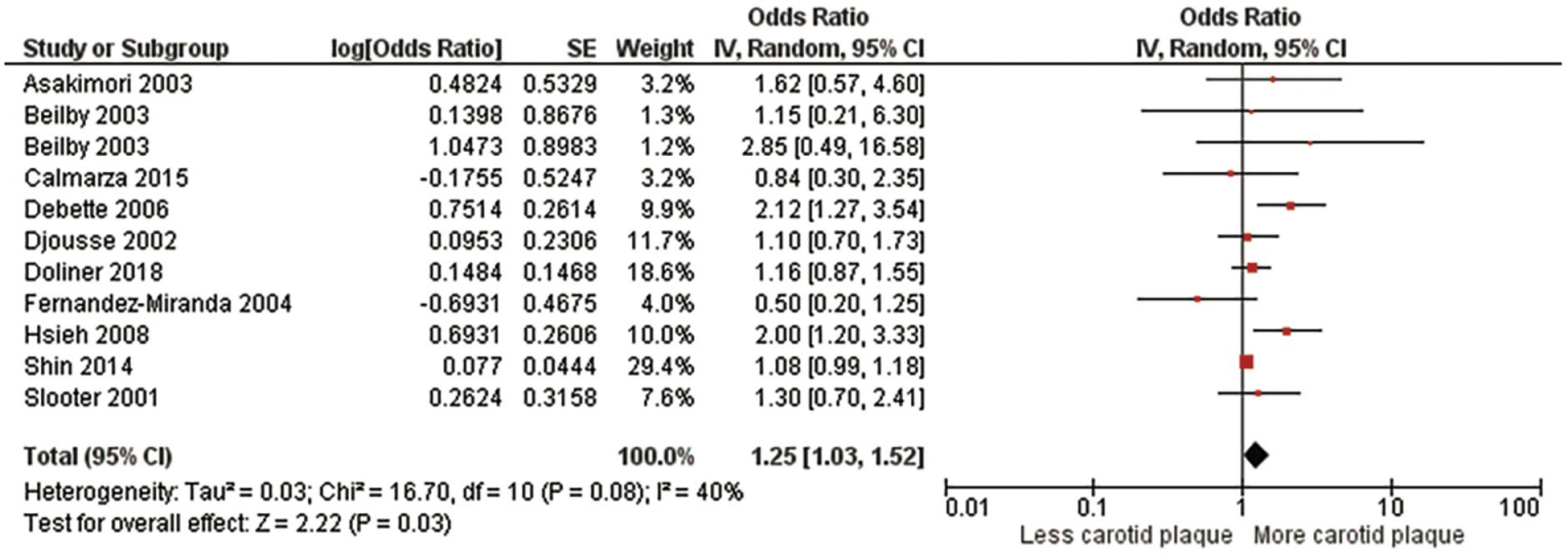
Association between the ϵ4 allele and carotid plaque.

A second meta-analysis evaluated those who had at least one *ε*4 allele which included homozygotes and heterozygotes (Figure 3, Supplementary Figure S2). This meta-analysis included 34,392 subjects from 10 studies (5, 12, 13, 16, 25–27, 29, 32, 33), the pooled OR was 1.25 (95% CI:1.03, 1.52), *p *= .03). There was no significant heterogeneity, Chi^2^, 16.70; *I*^2^, 40%; (*p *= .08). After sensitivity analysis, again, findings did not change the study's original conclusion (*p* values ranged from <.009 to.07).

**Figure 4:**
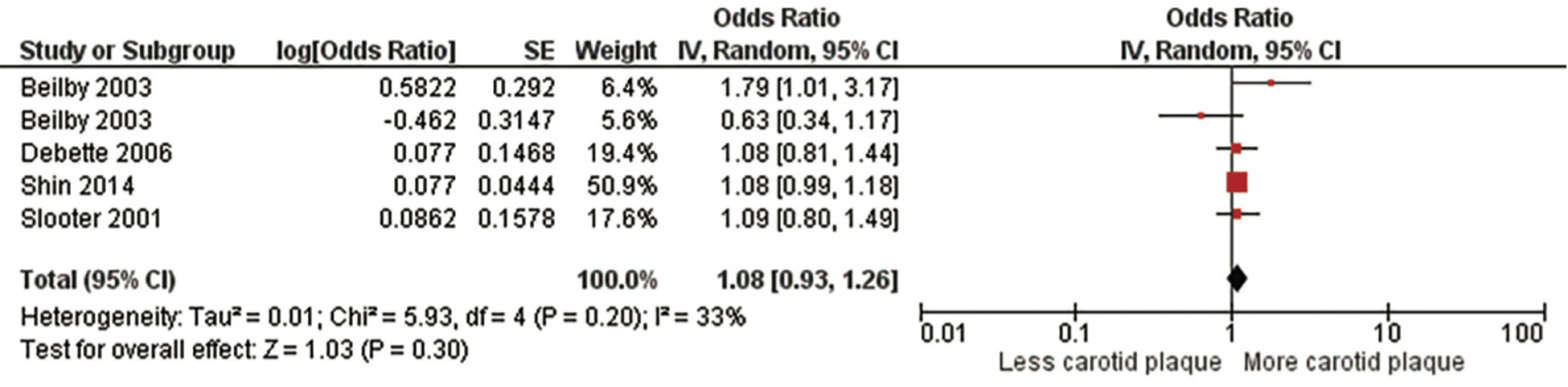
Association between ϵ4 heterozygotes and carotid plaque.

Lastly, we performed a meta-analysis of subjects who were heterozygous for the *ε*4 allele (*ε*3/ε4) including 31,567 subjects from four studies (5, 12, 32, 33) (Figure 4, Supplementary Figure S3). There was no significant association with a pooled OR of 1.08 (95% CI:0.93, 1.26), *p* = .30). There was no significant heterogeneity evident, Chi^2^, 5.93; *I*^2^, 33%; (*p *= .20). We found no significant predictors of between study heterogeneity in univariable meta-regression models (Supplementary Table S5).

### Assessment of risk of bias and publication bias for the meta-analysis studies

All studies were deemed suitable for inclusion with a low risk of bias (Supplementary Table S6). Visual inspection of each funnel plot (Supplemental images S1–S3) for each analysis appeared symmetrical. After, quantitative analysis with Egger's regression no evidence of publication bias was found (*p*-values >.99).

## Discussion

This systematic review and meta-analysis examined the literature for an association between APOE polymorphisms and carotid plaque. The findings showed a significant association between the *ε*4 allele and the presence of carotid plaque. The strongest association was present between *ε*4 homozygote individuals and carotid plaque. The association, however, was also significant for those individuals with at least one *ε*4 allele (both homozygotes and heterozygotes). There was no significant association between those with only one *ε*4 allele (heterozygotes) and the presence of carotid plaque, indicating the strength of association decreases in those with only one *ε*4 allele compared with those with both alleles. These findings suggest that the presence of an *ε*4 allele may play an important contributory role in the development of atherosclerotic plaque in the carotid arteries.

APOE plays a central role in determining plasma levels of cholesterol and hyperlipidemia and has been investigated as a key determinant of atherosclerosis. The *ε*4 allele has important genetic implications given the associated higher serum total and LDL cholesterol than the *ε*2 or *ε*3 alleles (37). Furthermore, the *ε*4 allele has been shown as a significant genetic risk factor for coronary artery disease (38, 39). The present study's findings are compatible with other studies which demonstrated an association between C-IMT and the APOE genotype (7). A prior study showed that *ε*4 carriers had elevated C-IMT independent of vascular risk factors or demographics (13). Both C-IMT and carotid plaque are frequently used as imaging biomarkers of atherosclerosis. Carotid plaque, however, is a more advanced form of atherosclerosis and as such was beneficial to examine this marker of disease. At the time of writing, to the author's knowledge, this is the first systematic review to examine the association between APOE and carotid plaque.

Plaque formation is an important manifestation of atherosclerosis, and the presence of carotid plaque helps to predict future cardiovascular events (40). This study examined carotid plaque because it is a stronger predictor of cardiovascular risk than C-IMT (41, 42) thereby the findings would have more clinical relevance in identifying those at risk of future ischemic events. The relationship between C-IMT and atherosclerotic plaque has been debated (40, 43). C-IMT is thought to constitute more than one morphological process and studies suggested it could represent adaptive changes to increased shear stress with aging rather than solely atherosclerotic changes (44). However, studies of the general population found that elevated C-IMT thickness predicted the later development of carotid plaque in individuals without plaque at baseline (45). Carotid plaque is thought to primarily reflect atherosclerosis as plaque begins in the subintima layer (46). Cervical carotid plaque is used as a marker and measure of atherosclerosis along with a risk predictor for future ischemic events (47).

There are several limitations to this study. Firstly, the authors did not search the gray literature and studies without an English translation were not assessed which potentially could introduce some publication bias. The authors acknowledge the heterogeneity in the measurement and assessment of carotid atherosclerotic plaque. The definition was not uniformly defined; however, the majority of studies used the Manheim C-IMT Consensus to define plaque (plaque is defined as a focal structure that encroaches into the arterial lumen of at least 0.5 mm or 50% of the surrounding IMT value or demonstrates a thickness of ≥1.5 mm) (48). Specifying a numerical cut-off for carotid plaque helped to reduce the subjectivity of plaque reporting. Future prospective studies would overcome this limitation with standardized plaque assessments using a pre-defined consensus guideline such as the Mannheim Consensus to measure plaque. Such standardization will homogenize data, facilitate future collation and comparison of results from different studies, and enable additional meta-analysis.

Plaque comparisons were based on a single imaging modality, ultrasound evaluation. While this permitted greater ease of study comparisons, detailed reporting on plaque morphology was lacking. Non-invasive imaging can readily characterize plaque features, specifically evaluating features of vulnerability and stability (14, 15, 49). For example, one of the included studies reported the *ε*2 allele was an independent risk factor for vulnerable plaque (28). Future studies could incorporate multimodality plaque assessments to provide a more comprehensive plaque assessment, including size, volume, morphological features, and overall plaque stability and vulnerability. Incorporating additional imaging data with the genetic assessment would provide a more comprehensive understanding of the relationship between APOE and carotid plaque. This would advance our knowledge beyond the presence or absence of plaque. Studies including plaque size and volume could enhance our understanding of carotid plaque phenotypes which may be beneficial when predicting cardiovascular risk (40).

Finally, there was some deviation in how studies reported the *ε*4 allele. Some studies combined both heterozygous and homozygous individuals. Because of this variation, three separate meta-analyses were performed to evaluate the different combinations of alleles. Future studies could overcome this limitation by specifying the results of the *ε*4 allele assessment separately according to an individual's allele status, dividing them into either homozygotes or heterozygotes. This would improve the accuracy of future analysis and permit greater ease of comparison of studies.

This study has important implications for practice. Carotid atherosclerosis is a recognized major risk for stroke. The genetic determinants of carotid plaque and plaque morphology remain unclear. This meta-analysis highlights the importance of delineating the role of genetic variants in carotid atherosclerotic disease. Continued research is warranted to validate this association. The results of this meta-analysis could stimulate further studies attempting to provide a greater understanding of APOE phenotypes and their possible carotid atherosclerotic phenotypes. While these results may not immediately change clinical practice, they highlight the necessity to understand the role of genetic determinants of atherosclerosis.

## Conclusion

This systematic review and meta-analysis suggest an association between the APOE E4 genotype, predominantly for *ε*4/ε4 homozygotes and the presence of carotid atherosclerotic plaque. If this association between the E4 genotype and carotid atherosclerotic plaque is confirmed, then the E4 genotype may play a contributory role in the development of ischemic stroke. Future prospective research evaluating the relationship between plaque morphology and APOE polymorphisms would be highly beneficial.

## Data Availability

The original contributions presented in the study are included in the article/**Supplementary Material**, further inquiries can be directed to the corresponding author.
