# Supplementary material for: Extracranial carotid artery atherosclerotic plaque and APOE polymorphisms: a systematic review and meta-analysis
Source: Front Cardiovasc Med. 2023 Nov 13;10:1155916. doi: 10.3389/fcvm.2023.1155916 (PMC10683092; doi:10.3389/fcvm.2023.1155916)
Supplement: Supplementary file 1 [file Datasheet1.pdf]

Supplemental Material

Search Strategy

1. carotid artery diseases/ or carotid stenosis/
2. carotid artery disease/ge or carotid stenosis/ge
3. carotid arteries/
4. (carotid adj5 [atherosclero$ or arteriosclero$ or steno$ or plaque or sclero$ or atheroma$ or disease$ or disorder$]).tw.
5. 1 or 3 or 4
6. apolipoproteins/ or apolipoproteins e/
7. ([apolipoprotein$ adj e] or [apoprotein$ adj e] or apo-e or apo e or apoe).tw.
8. 6 or 7
9. 5 and 8
10. 2 or 9
11. limit 10 to humans

Supplemental Images

Supplemental Image S1: Funnel Plot of the Association between ε4/ε4/ Homozygotes and Carotid Plaque

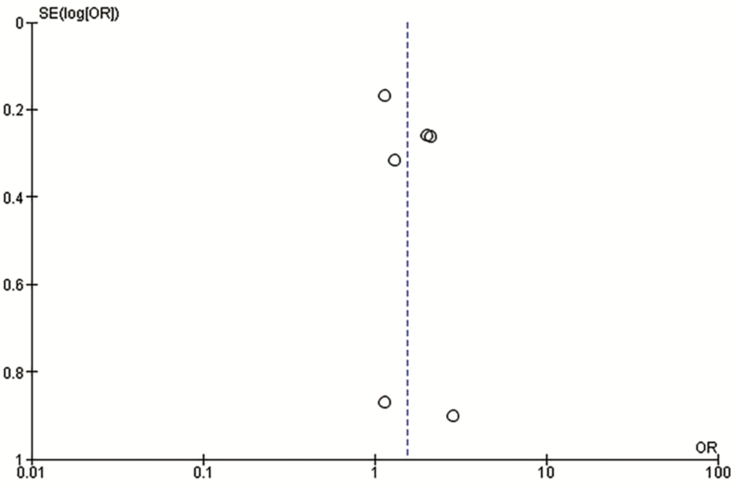

Supplemental Image S2: Funnel Plot of the Association between ε4/ε4/ Homozygotes and Carotid Plaque

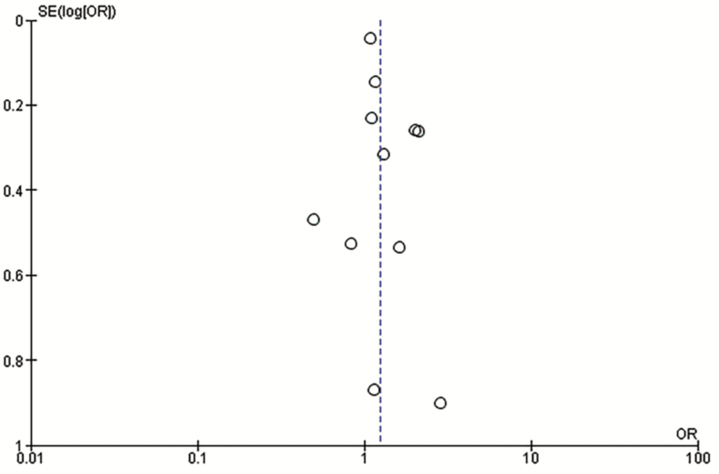

Supplemental Image S3: Funnel Plot of the Association between ε4 Heterozygotes and Carotid Plaque

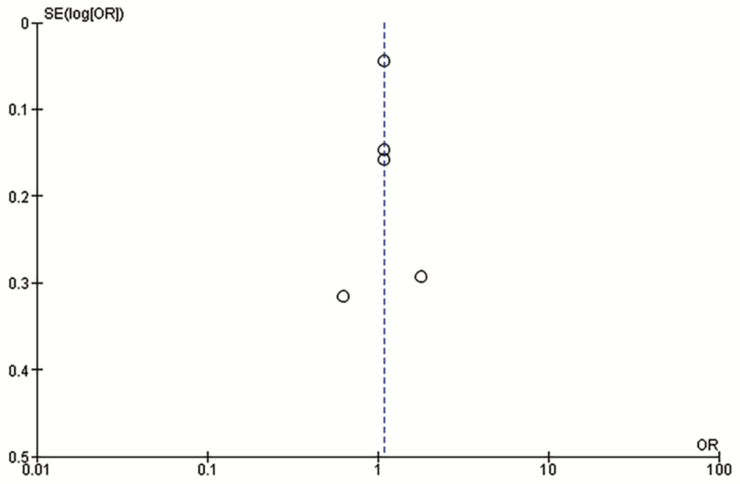

Supplemental Tables

Table S1: Patient Characteristics

Primary Author	Year	Country	No of Centers	Study Design	Study Type	Inclusion Criteria	Exclusion Criteria	Population Description
**Asakimori**	2003	Japan	Single	Cross-sectional	Prospective	Chronic renal failure on hemodialysis	Diabetic nephropathy	Outpatient hemodialysis patients
**Beilby**	2003	Australia	Single	Cross-sectional	Prospective	Random selection of community subjects		Randomly selected Perth Community, equal man-to-woman ratio and equal numbers in each age decile between 20 and 70 years
**Blazejewska-Hyzorek**	2014	Poland	Single	Cross-sectional	Prospective	Stroke, within 7 days from admission		All consecutive ischemic stroke patients, admitted within 7 days from stroke onset, two-year study period
**Bleil**	2006	US	Single	Cross-sectional	Prospective	From the University of Pittsburgh Reactivity and Cardiovascular Risk Trial (REACT)		Untreated hypertensive white men, confirmed by two resting blood pressure measurements averaging 140–180 mmHg SBP or 90–110 mmHg DBP on each of two evaluations

**Calmarza**	2015	Spain	Single	Cross-sectional	Prospective	Over 40 years and their age and gender distribution matched the 2004 - 2010 Spanish population census	Major cardiovascular events (myocardial infarction, stroke, and limb amputation due to peripheral artery disease)	Randomly sele[illegible] from the regist[illegible] the 200,000 re[illegible] population of "Gamonal Ant[illegible] Health Care C[illegible] in Burgos, Spa[illegible]
**Debette**	2006	France	Multi	Cross-sectional	Prospective	Aged 65 years and over	Nonnative French speakers were dropped	Recruited from electoral rolls [illegible] French cities F[illegible] native speakers[illegible]
**Djousse**	2002	US	Multi	Cross-sectional	Prospective	592 families randomly selected, and 661 families chosen because of higher-than-expected CHD rates among family members.		Families who [illegible] randomly sele[illegible] and families ch[illegible] because of hig[illegible] than-expected [illegible] rates among fa[illegible] members
**Doliner**	2018	US	Single	Population-based cross-sectional	Prospective	A subsample of participants of the Northern Manhattan Study (NOMAS), an ongoing prospective population-based study of stroke incidence and vascular risk factors	No baseline history of stroke, myocardial infarction, or chronic inflammatory conditions such as systemic lupus erythematosus, Lyme disease, gonococcal arthritis, or	Subsample of Northern Man[illegible] Study (NOMA[illegible] enrolled in Oral Infectious Disease Vascu[illegible] Disease Epidemiology (INVEST) asse[illegible] for the purpose[illegible] both studies

							bacterial endocarditis	
**Fernandez-Miranda**	2004	Spain	Single	Cross-sectional	Prospective	Consecutive patients with coronary disease referred to Lipid and Atherosclerosis Unit		Consecutive pa with coronary disease referre Lipid and Atherosclerosi
**Hsieh**	2008	Taiwan	Single	Cross-sectional	Prospective	Random sample of 479 subjects of 235 cases and 244 controls genotyped of APOE and MCP-1 were selected without any matched factors		8088 residents ≥40 years from villages in four townships were interviewed an included in the cohort during t years 1991–19 1318 cohort members who agreed to partic in this study fi the health examination including the ultrasonograph assessment. Th study was a ran sample of 479 subjects genoty
**Kahraman**	2004	Turkey	Single	Cross-sectional	Prospective	Stable renal function at least 6 months after transplantation	History of diabetes, atherosclerotic vascular disease (previous myocardial	Leading a norm active life post transplant. Not drugs affecting lipoprotein metabolism

							infarction, angina, revascularization procedures, stroke, or transient ischemic attack) and smoker	
**Karvonen**	2002	Finland	Single	Cross-sectional case-control	Prospective	300 hypertensive men and 300 control men living in the City of Oulu were randomly selected by age stratification (15 subjects/year) from population registers		Male cohort of OPERA study, randomly selec from the Socia Insurance Insti register for reimbursement antihypertensiv
**Lambrinoudaki**	2008	Greece	Single	Cross-sectional	Prospective	Menopausal for at least 1 year, confirmed by FSH and estradiol	No clinically overt cardiovascular disease, thromboembolism, diabetes mellitus, untreated thyroid dysfunction, history of gynecological malignancy	Recruited from Menopause Cl Criteria for inc were absence o climacteric symptoms, endometrial thickness ≤5m history of gynecological malignancy, clinically overt cardiovascular disease, thromboembol diabetes mellit

								untreated thyro dysfunction, an treatment with lowering or antihypertensiv medications
**Shin**	2014	Korea	Multi	Cross-sectional	Prospective	Middle age and Elderly Koreans Dong-gu study 50 or older identified from 2005 census		Two cohort stu the Namwon S and the Dong-g Study investiga determinants o occurrence and progression of cardiovascular diseases, osteoporosis, dementia, and cancer among middle-aged or elderly Korean
**Slooter**	2001	Netherlands	Single	Population-based, single-center cohort study	Prospective	All inhabitants of a suburb of Rotterdam, aged at least 55 years, including people living in homes for the elderly, were invited		Aged at least 5 years living in suburb Ommor study on chron disabling disea
**Viticchi**	2014	Italy	Single	Cross-sectional	Prospective	Diagnosis of mild cognitive impairment according to the National Institute on Aging and the Alzheimer's	Basal MMSE score <24 adjusted according to age and education as assessed for the Italian population, b)	Consecutive su referred to dem outpatient serv by GPs for suspected cogn impairment ove year period. Included only

						Association diagnostic criteria	focal neurological signs at physical examination, c) presence of prior significant general or neurological conditions, d) history of acute cerebrovascular or cardiac events, e) severe leukoencephalopathy, and f) major psychiatric pathologies	subjects withou[illegible] vascular lesion[illegible] (grade 0) or wi[illegible] small subcortic[illegible] focal lesions d[illegible] as areas of hig[illegible] signal intensity[illegible] T2-weighted i[illegible] but isointense [illegible] normal brain o[illegible] weighted imag[illegible] (grade 1)
**Zurnić**	2014	Serbia	Single	Cross-sectional	Prospective	Consecutive patients with carotid atherosclerosis who underwent carotid endarterectomy	Carotid kinking, carotid aneurysm, history of previous carotid endarterectomy, tumors, autoimmune disease, chronic inflammatory diseases, renal failure	Caucasians of European descendent fro[illegible] Serbia, 285 co[illegible] 210 patients

Table S1: Patient Characteristics Continued

Primary Author	Subjects N	Age Mean (SD) Years	Males N (%)	Females N (%)	ε4 allele frequency N or %	BMI Mean (SD) Kg/m^2	Diabetes N (%)	Hypertension N (%)
**Asakimori**	163	54.5	85 (52.1)	78 (47.9)	32<br>ε2/4=1,<br>ε3/4=31	N/S	Excluded	82 (50.3)<br>26 (59.1) with plaque<br>56 (47.1) without plaque
**Beilby**	1109	52.5*<br>52 (13) Males<br>53 (13) Females	558 (50.3)	551 (49.7)	304<br>ε4=14.7%<br>ε2/4=21<br>ε3/4=262<br>ε4/4=21	26.1 (4.1)*	N/S	266 (24.0)<br>135 Males<br>131 Females
**Blazejewska -Hyzorek**	388	68 (11.1)	203 (52.3)	185 (47.7)	71<br>ε3/4 69<br>ε4/4=2	N/S	73 (19.0)	257 (66.8)
**Bleil**	182	56 (8.9)	182 (100.0)	0 (0)	ε4=12.6%	28.3 (3.1)	N/S	182 (100.0)
**Calmarza**	171<br>Apo E 157	64.2 (12.0)	89 (52.1)	82 (47.9)	30 (19.1%)	28.1 (3.7)	N/S	N/S
**Debette**	5856	73.5 (4.9)	2319 (39.6)	3537 (60.4)	1216<br>ε 4 allele=11.0%<br>ε2/4=92<br>ε3/4=1053<br>ε4/4=71	24.9 (3.7)	539 (9.2)	3596 (61.4)
**Djousse**	544	56.4 (10.1)	245 (45.0)	299 (55.0)	140<br>ε4=14%<br>ε3/4=130<br>ε4/4=10	27.9 (5.5)*	N/S	365 (67.1)

**Doliner**	1243	69 (9.0)	478 (38.0)	765 (62.0)	309<br>ε4=14%	28.0 (5.0)<br>28.0 (5.0)<br>Males<br>28.0 (5.0)<br>Females	153 (12.0)	492 (40.0)
**Fernandez-Miranda**	225	60.9 (7.8)	191 (84.9)	34 (15.1)	49<br>ε4=22%	25.9*<br>E2 25.3 (2.5)<br>E3 26.2(3.4)<br>E4 26.0 (2.8)	49 (21.8)	108 (48.0)
**Hsieh**	479	Mean N/S<br>≥40-55=96<br>subjects<br>56-65=201<br>≥66=182	224 (46.8)	255 (53.2)	89<br>ε4=36 controls<br>ε4=53 cases	N (%)<br>181(37.8)	72 (15.0)	135 (28.2)
**Kahraman**	118	40 (8.0)	80 (67.8)	38 (33.2)	14<br>E4=12%	26.0 (4.0)	N/S	N/S
**Karvonen**	511	50.9	511<br>(100.0)	0	176<br>ε4=18.9%	28.0*<br>ε4=29.3 (4.3)<br>hypertensives<br>ε4=25.5 (3.8)<br>controls	443 (86.7)	N/S
**Lambrinoudaki**	84	57.7 (4.9)	0 (0)	84 (100.0)	N/S	26.8 (4.4)	N/S	N/S
**Shin**	19201<br>Dong-gu<br>study<br>9056<br>Namwon<br>study<br>10,145	63.3*<br>Dong-gu<br>65.2 (8.2)<br>Namwon<br>61.6 (7.8)	7627<br>(39.7)<br>Dong-gu<br>3625 (40)<br>Namwon<br>4002<br>(39.5)	11574<br>(60.3)<br>Dong-gu<br>5431 (60)<br>Namwon<br>6143<br>(60.5)	E4=9%<br>combined<br>cohort<br>Dong-gu<br>E4/4= 84<br>E3/E4=1413<br>E2/E4=118<br>Namwon<br>E4/4=88<br>E3/E4=1484<br>E2/E4=107	24.4*<br>combined<br>Dong-gu<br>24.4*<br>Namwon<br>24.4*	2968 (15.5)<br>Dong-gu<br>1728 (19.1)<br>Namwon<br>1240 (12.3)	8076 (42.1)<br>Dong-gu<br>4052 (44.7)<br>Namwon<br>4024 (39.7)

**Slooter**	5401	69.2*<br>E2/E4 69.2 (8.7)<br>E/3E4 69.1 (8.8)<br>E4/E4 61.8 (7.6)	2202 (40.8)	3199 (59.2)	1529<br>E2/E4=137<br>E3/E4=1258<br>E4/E4=134	26.3*<br>E2/E4=26.5 (3.5)<br>E3/E4=26.1 (3.7)<br>E4/E4=26.1 (3.6)	544 (10.1)	N/S
**Viticchi**	75	74.4*<br>ε4+=72.91 (7.68)<br>ε4-=75.14 (5.79)	26 (34.7)	49 (65.3)	24 ε4+	N/S	9 (12.0)	48 (64.0)
**Zurnić**	495	55.1*<br>51.5 (9.3) healthy controls<br>60.02 (10.08) patients	265 (53.5)	230 (46.5)	81<br>E2/4=5<br>E3/4=71<br>E4/4=5	N/S	N/S	N/S

Table Notes: *Indicates weighted pooled means**,** N/S=Not specified

Table S1: Patient Characteristics Continued

Primary Author	Total Cholesterol /Dyslipidemia Mean (SD)/ N (%)	Smoking History N (%)	TIA/Stroke N (%)	CVD/MI N (%)	Carotid Plaque N (%)
**Asakimori**	N (%) 66 (40.5)*<br>N=16 with plaque<br>N=50 without plaque	47 (28.8)	N/S	N/S	44 (27.0)<br>31 Males<br>13 Females
**Beilby**	5.6 (1.0) mmol/L*<br>5.5 (1.0) mmol/L Males<br>5.6 (1.0) mmol/L Females	16.5 pk-yr*<br>16.7 pk-yr Males<br>7.9 pk-yr Females	22 (2.0)<br>Strokes<br>11 Males<br>11 Females	N/S	284 (25.6)<br>161 Males<br>123 Females
**Blazejewska -Hyzorek**	N (%) 207 (55.5) > 200mg/dL	192 (49.5)	103 (26.6)<br>54 (14.0)<br>Strokes<br>49 (12.7) TIA	114 (29.5)	223 (57.4)
**Bleil**	N/S	106 (58.2)	N/S	N/S	125 (68.7)
**Calmarza**	5.53 (1.06) mmol/L	57 (33.5)	N/S	18 (10.5)	41 (24)
**Debette**	5.8 (1.0) mmol/L	2301 (39.3)	855 (14.6)<br>Strokes and/or MI	855 (14.6)<br>Stroke and/or MI	43<br>62 % of<br>ε4/4=71
**Djousse**	LDL 3.4 (0.9) mmol/L for E4 (ε4/4 or ε3/4)	76 (14.0)	N/S	N/S	ε4 allele 31/128 smokers<br>ε4 allele 24/65
**Doliner**	LDL 128 (35.0) mg/dL	636 (51.7)	N/S	N/S	682 (55)<br>178 (58) ε4 carriers
**Fernandez-Miranda**	5.8 mmol/L<br>5.3 (1.1) mmol/L E2<br>5.8 (1.0) mmol/L E3<br>5.9 (1.0) mmol/L E4	47 (20.9)	N/S	13 (5.8) Family Hx	N/S

**Hsieh**	≥ 200 mg/dL N (%) 248 (51.8)<br>126 control group<br>122 cases	175 (36.5)	N/S	N/S	479<br>ε4 36 cases<br>ε4 53 controls<br>Other APOE 208 cases, 182 controls
**Kahraman**	221 (41) mg/dL	N/S	N/S	N/S	39 (33.1)
**Karvonen**	5.8 mmol/L*<br>5.93 (1.0) mmol/L E4 hypertensives<br>5.86 (0.97) mmol/ E4 controls	358 (70.1)	N/S	N/S	Mean of plaques 2.5 (ε4 present)<br>1.8 (ε4 absent)
**Lambrinoudaki**	N/S	28 (33.3)	N/S	N/S	APO E2/E3/E4<br>Wild type 33.3%<br>Heterozygotes 25%<br>Homozygotes 16.7%
**Shin**	195.4 mg/dL combined<br>Dong-gu 201.3 mg/dL<br>Namwon 189.4 mg/dL	2559 (13.3)<br>Dong-gu 992 (11.0)<br>Namwon 1567 (15.5)	734 (7.2)<br>Strokes<br>Dong-gu 367 (4.1)<br>Namwon 367 (3.6)	157 (0.8) Dong-gu 119 (1.3)<br>Namwon 38 (0.4)	N/S
**Slooter**	6.6 mmol/L*<br>E2/E4=6.54 (1.1) mmol/L<br>E3/E4=6.62 (1.21) mmol/L<br>E4/E4= 6.81 (1.04) mmol/L	1219 (22.6)	N/S	N/S	N/S
**Viticchi**	N (%) 36 (48)	14 (18.7)	N/S	N/S	26 (34.7)<br>ε4+=13<br>ε4-=13 IMT >1 mm
**Zurnić**	3.74 (1.09) mmol/L E3/E4 and E4/4	N/S	N/S	N/S	210 (42.4)

Table S2: Imaging Assessment

Primary Author	Modality	Type of Diagnostic Ultrasound System	Transducer	Mode	Definition of Plaque	Type of Operators	No Operators	Clinical Blinding
**Asakimori**	US	SSD 2000; Aloka, Tokyo, Japan	7.5 MHz	High-resolution real-time scanner	A maximum IMT of 1.5 mm or greater was considered plaque	Trained physician	1	Y
**Beilby**	US	Interspec (Apogee) CX 200	7.5-MHz annular phased-array transducer	B mode	Clearly identified area of focal increased thickness (≥1 mm) of the intima-media layer	Trained sonographers (2)	2	N/S
**Blazejewska -Hyzorek**	US	Acuson 128XP/10C, Siemens,Berlin and Munich, Germany	7 MHz	B mode	Localized echo structures encroaching into the vessel lumen for which the thickness was ≥1 mm	Sonographer	1	N/S
**Bleil**	US	Toshiba SSA-270 scanner;Nasu, Japa	N/S	B mode	Distinct area of hyper echogenicity and/or a focal protrusion into the lumen of the vessel	Sonographers	N/S	N/S
**Calmarza**	US	HP lmage Point equipment	10 MHz linear probe	B mode	A hyper-echogenicity or any protrusion	Investigator	1	Y

					of the intima-media in the vascular lumen of at least twice the thickness of the adjacent segment			
**Debette**	US	Ultramark 9 High-Definition Imaging	5-10 MHz	B mode	Localized echo structures that encroached into the vessel lumen and for which the distance between the media-adventitia interface and the lesion surface facing the lumen was ≥1 mm	Sonographers	N/S	N/S
**Djousse**	US	Biosound Phase 2 system	10 MHz	B mode	Based on the ultrasound images, it was determined whether an atherosclerotic lesion was present in the segment visualized	Trained sonographers	N/S	N/S
**Doliner**	US	GE LogIQ 700	9-13 MHz linear array	B mode	Focal wall thickening or protrusion in the lumen more than 50% greater than the surrounding	Trained and certified sonographers	N/S	N/S

					thickness. Carotid plaque areas were measured with the automated computerized edge tracking software program M'Ath (M'Ath Inc, Paris, France)			
**Fernandez-Miranda**	US	Toshiba	7.5 MHz color doppler transducer		Focal widening of the vessel lumen 50% greater than IMT of neighboring sites, or an IMT >1.2 mm	Radiologist	1	N/S
**Hsieh**	US	Hewlett-Packard SONO 100	7.5 MHz	B mode and a 5.6-MHz pulsed-Doppler mode scanner	Diagnosed as having prevalent asymptomatic carotid atherosclerosis by a neurologist based on a mean carotid IMT of >1.0 mm and either plaque occurrence in at least two locations on one side (right or left) or the occurrence of stenosis of	Neurologist	1	Y

					>50% in the left or right CCA			
**Kahraman**	US	Toshiba SSA-270 A; Toshiba, Tokyo, Japan	7.5 MHz	High-res B-mode	Protrusion into the vascular lumen more than 1.5 mm	Experienced physician	1	Y
**Karvonen**	US	Toshiba SSA-270A; Toshiba Corp, Tokyo, Japan	7.5 MHz	B mode, pulsed doppler, color mode	Echogenic structure encroaching into vessel lumen and IMT 50% greater than neighboring sites	Trained radiologist	1	Y
**Lambrinoudaki**	US	N/S	N/S	B mode	A clearly identified area of focally increased IMT greater than 1.2 mm	N/S	N/S	N/S
**Shin**	US	SONOACE 9900, Medison, Korea	7.5 MHz electric linear array	High-res B-mode	Focal structures that encroached into the lumen by at least 100% of the surrounding IMT value	Sonographers	N/S	Y
**Slooter**	US	ATL UltraMark IV	7.5 MHz linear array	B-mode	Focal widening relative to adjacent segments with protrusion into the lumen composed of either only calcified	N/S	N/S	N/S

					deposits or a combination of calcification and noncalcified material. No attempt was made to quantify the size or extent of the lesions			
**Viticchi**	US	iU22 Philips Ultrasound, Bothell WA	7.5 MHz	Echo-Color-Doppler	Mannheim Consensus, a focal structure protruding into the arterial lumen of at least 0.5 mm or 50% of the surrounding IMT value or thickness >1.5 mm measured from the media-adventitia interface to the intima-lumen interface	N/S	N/S	N/S
**Zurnić**	US	Acuson Antares™ system, Siemens, Munich, Germany	5-12 MHz linear array	B mode, duplex	Focal widening relative to adjacent segments as evidenced by protrusion into the lumen and/or localized roughness with	N/S	N/S	N/S

					increased echogenicity. Carotid atherosclerosis was defined as the presence of atherosclerotic plaques in the internal or common carotid artery			

Table S2: Imaging Assessment Continued

Primary Author	Coverage	Intraobserver Variability	Interobserver Variability	Description of Study Readers
**Asakimori**	CCAs bilaterally to carotid bifurcation in 3 longitudinal projections and transverse projections	Intraobserver variability of IMT measurement, coefficient of variation was 2.0%	N/S	All subjects were examined by a single trained physician

		30 subjects examined on separate occasions 7-14 days apart		blinded to their clinical characteristics
**Beilby**	Distal CCAs, Carotid bulbs, ICAs, ECAs	Intraobserver coefficients of variability were 2.9% for sonographer 1 and 4.8% for sonographer 2	Interobserver coefficient of variability was 5.9%	N/S
**Blazejewska -Hyzorek**	CCAs, Carotid bifurcations, origin (first 2 cm) of ICAs and ECAs	Intraobserver coefficients of variability were 4.0% for performing sonographer	N/S	N/S
**Bleil**	CCAs, Bifurcations, and the first 1 cm of ICAs	N/S	Intraclass correlation for the plaque index was 0.93. 15 participants had carotid scans performed by 2 sonographers on 2 occasions approximately 2 weeks apart<br>read by 2 readers	Used a modified computerized reading program developed for the Cardiovascular Health Study
**Calmarza**	CCAs, Bifurcations, ECAs and ICAs accessible to ultrasound	N/S	N/S	Measurements were performed blind to the rest of the data by the same investigator who had previous experience in making these measurements
**Debette**	CCAs, Bifurcations, Origin of ICAs	N/S	N/S	Centralized reading protocol
**Djousse**	CCAs (1 cm proximal to the dilatation of the carotid bulbs), bifurcations (1cm segment proximal to the flow divider), and ICAs (1 cm	Estimates of the site-specific reliability coefficients were 0.77, 0.73, and 0.70 for mean carotid far-wall IMT at the carotid bifurcation, internal	Estimates of the site-specific reliability coefficients were 0.77, 0.73, and 0.70 for mean carotid far-wall IMT at	Trained readers<br>Centralized reading protocol

	segment in the internal branch distal to the flow divider)	carotid artery, and common carotid artery, respectively	the carotid bifurcation, internal artery, and common carotid artery, respectively	
**Doliner**	CCAs, Bifurcations, ICAs	N/S	N/S	Standardized scanning and reading protocols by trained and certified sonographers
**Fernandez-Miranda**	CCAs, Bifurcations, origin of ICAs	N/S	N/S	All ultrasonographic assessments of carotid arteries performed by the same radiologist
**Hsieh**	CCAs, ICAs, ECAs	N/S	N/S	All measurements were bilateral and performed by one neurologist blinded from the clinical details
**Kahraman**	CCAs, Bifurcations	N/S	N/S	All examinations were carried out by the same experienced physician, unaware of patient history and laboratory findings
**Karvonen**	CCAs, Bifurcations, ICAs and ECAs as far as possible	Intrareader variability and correlation coefficient (Pearson) were 3% and 0.97 for mean IMT. Assessed from 30 randomly subjects by two radiologists blind to original results	Interreader variability and correlation were 7.2% and 0.93	Measurements were made from the video image viewed on the monitor of the ultrasound device using electronic calipers
**Lambrinoudaki**	CCAs, Carotid bulbs, first 1cm ICAs	N/S	N/S	N/S
**Shin**	CCAs, Bifurcations, ICAs	N/S	N/S	A single trained physician reader

				analyzed the frozen images using SigmaScan Pro Version 5.0.0(SPSS Inc., Chicago, IL, USA) according to a standardized protocol
**Slooter**	CCAs, Bifurcations, ICAs	N/S	N/S	N/S
**Viticchi**	CCAs, Carotid bulbs, ICAs	N/S	N/S	N/S
**Zurnić**	CCAs, ICAs	Intrarater reliability intraclass coefficient was 0.968	Interrater reliability was 0.916	N/S

Table Notes: CCA= common carotid artery**,** ECA= external carotid artery, ICA= internal carotid artery

Table S3: Genetic Testing

Primary Author	Year	Genotyping Method	DNA Extraction Method	Restriction Enzyme	Electrophoresis Description	Visualized	Readers	Base-Pair Fragment Amplified /Primers
**Asakimori**	2003	PCR/RFLP	Peripheral-blood mononuclear cells using a DNA purification kit (Wizard; Promega, Madison, WI).	HhaI	15% polyacrylamide nondenaturing gel	Ethidium bromide staining with UV transillumination	N/S	244-bp fragment Forward and reverse primers described
**Beilby**	2003	PCR	Extracted by the salt/phenol/chloroform method from the cells of the buffy coat	N/S	N/S	N/S	N/S	Two primers sense and anti-sense oligo

**Blazejewska-Hyzorek**	2014	PCR/RFLP	Extracted from the whole frozen EDTA-blood using the TRI Reagent (SIGMA, Poznan, Poland)	HhaI	8% polyacrylamide nondenaturing gel	Treated with ethidium bromide and visualized with ultraviolet illumination	N/S	Primers described
**Bleil**	2006	PCR	N/S	HhaI	8% polyacrylamide nondenaturing gel	Treated with ethidium bromide and visualized with ultraviolet illumination	N/S	Primers described
**Calmarza**	2015	PCR	Extracted from EDTA anticoagulated whole blood with DNA extraction kit (DNA extraction kit, Genomic DNA Purification System, Promega, USA)	N/S	2% agarose	Added ethidium bromide	N/S	N/S
**Debette**	2006	PCR	Used fluorogenic allele-specific oligonucleotide probes (TaqMan assay)	HhaI	8% polyacrylamide gels (Invitrogen, Karlsruhe, Germany)	Stained with ethidium bromide solution and transillumination with ultraviolet light	N/S	Probes and primers described
**Djousse**	2002	PCR	N/S	HhaI	Separated by electrophoresis	Silver stained	N/S	267-bp fragment from exon 4 of the apoE gene
**Doliner**	2018	PCR	Two SNPs were genotyped using TaqMan® allelic	N/S	N/S	N/S	N/S	Two single-nucleotide polymorphisms

			discrimination assays (Applied Biosystems, Foster City, CA) following the manufacturer's instructions					(SNPs), rs429358 and rs7412
**Fernandez-Miranda**	2004	PCR	Genomic DNA was isolated from human leukocytes according to standard procedures	HhaI	Separated on Meta-Phor agarose, 2% agarose gels with Tris-borate-EDTA (TBE) buffer	N/S	N/S	244-bp fragment
**Hsieh**	2008	PCR/RFLP	DNA was extracted from the buffy coat using the Viogene Blood and Tissue Genomic DNA Miniprep System kit (Viogene Inc., Taipei, Taiwan)	HhaI	10% polyacrylamide gel	Stained with ethidium bromide	N/S	N/S
**Kahraman**	2004	PCR	Genomic DNA from leukocytes was purified according to the method of Miller et al. (Buffy coats of nucleated cells obtained from anticoagulated blood (ACD or EDTA) resuspended in	Cfol	3% agarose gel	Ethidium bromide staining	N/S	Primers described

			15 ml polypropylene centrifugation tubes with 3 ml nuclei lysis buffer (10 mMTris-HCl, 400 mM NaCl, 2 mM Na2EDTA, pH 8.2)					
**Karvonen**	2002	Isoelectric focusing and immunoblotting techniques	N/A	N/A	N/A	N/A	N/S	N/A
**Lambrinoudaki**	2008	PCR	Isolated from 200 microL of whole blood treated with ethylenediaminetetraacetic acid using the blood spin protocol of a QIAamp DNA Blood Mini Kit from Qiagen Corporation	N/S	N/S	N/S	N/S	N/S
**Shin**	2014	PCR	Extracted from peripheral blood with the AccuPrep Genomic DNA Extraction Kit (Bioneer, Seoul, Korea) or the QIAamp DNA Mini Kit (Qiagen	HhaI	10% polyacrylamide gels	Using ultraviolet illumination	N/S	A 244-bp fragment of the APOE gene that spanned the two polymorphic sites at codons 112 and 158

			Inc., Chatsworth, CA, USA) according to the manufacturer's protocol					
**Slooter**	2001	PCR	N/S	HhaI	Separated on precast gels (ExcelGel, Pharmacia Biotech, Uppsala, Sweden) by electrophoresis (MultiPhorII, Pharmacia Biotech) for 1½ hours at 600 V	Silver staining	3 Independent	Primers described. 227-bp region of DNA that spans both apo E polymorphic sites
**Viticchi**	2014	PCR	DNA extraction from peripheral blood samples by means of a nucleic acid isolation system (QuickGene-810, Fujifilm, Japan)	N/S	Automated rotary thermocycler (Rotor-Gene 6000, Corbett Research, Australia)	N/S	N/S	Primers described, 244 bp
**Zurnić**	2014	PCR/RFLP	Genomic DNA was isolated from the whole blood samples collected with ethylenediamine tetra acetic acid (EDTA) by standardized BloodPrep® DNA Chemistry isolation kit	Hin6I	8% polyacrylamide gel run for 2 h in electric field of 12 V/cm	Gels stained with silver nitrate, GDS8000 gel documentation system (Ultraviolet Products Inc, Upland, USA)	N/S	Primers described

			(Applied Biosystems, Forester City, CA) on the ABI PRISM™ 6100 Nucleic Acid Prep Station (Applied Biosystems, Forester City, CA)					

Table S4: Odds Ratio Results Included in the Meta-Analysis

Primary Author	Year	OR E4	OR ε4/ε4	OR ε3/ε4
**Asakimori**	2003	1.62 (0.57-4.43)		
**Beilby**	2003		2.85 (0.49–16.57) Males<br>1.15 (0.21–6.31) Females	1.79 (1.01–3.17) Males<br>0.63 (0.34–1.20) Females
**Calmarza**	2015	0.839 (0.300-2.345)		
**Debette**	2006		2.12 (1.27 -3.53)	1.08 (0.93–1.25)
**Djousse**	2002	1.1 (0.7–1.9)		
**Doliner**	2018	1.16 (0.87- 1.54)		
**Fernandez-Miranda**	2004	0.5 (0.2–1.1)		
**Hsieh**	2008		2.0 (1.2-3.2)	
**Shin**	2014	1.08 (0.99-1.18)	1.14 (0.82-1.58)	1.08 (0.99-1.18)
**Slooter**	2001		1.3 (0.7- 2.2)	1.09 (0.8-1.2)

Table Notes: OR= Odds Ratio

Table S5: Heterogeneity Assessment

	Meta-Analysis								
**Possible Source of Heterogeneity**	**Homozygotes (ε4/ε4) [n=6 studies]**			**At least one ε4 allele [n=11 studies]**			**Heterozygotes (ε3/ε4) [n=5 studies]**		
	p[(a)]	I^2 [(b)]	R^2 [(c)]	p	I^2	R^2	p	I^2	R^2
Publication year	.49	10	2	.54	38	0	.96	49	0
Mean age	.28	0	100	.21	15	100	.94	49	0
% Male	.45	40	16	.50	52	0	.34	28	0
% Hypertension	.95	52	0	.98	57	0	.39	49	0
% Smoking	.14	0	100	.33	43	0	.74	27	0
% Diabetes	.57	23	0	.10	33	59	.41	0	0

Table Notes: (a) p value, (b) I^2 = % residual variation due to heterogeneity, (c) R2 = percent of between-study variance explained

Table S6: Assessment of Risk of Bias with the Joanna Briggs Institute Questionnaire

Questions	Asakimori 2003	Beilby 2003	Calmarza 2015	Debette 2006	Djousse 2002	Doliner 2018	Fernandez-Miranda 2004	Hsieh 2008	Shin 2014	Slooter 2001
Were the criteria for inclusion in the sample clearly defined?	+	+	+	+	+	+	+	+	+	+
Were the study subjects and the setting described in detail?	+	+	+	+	+	+	+	+	+	+
Was the exposure measured in a valid and reliable way?	+	+	+	+	+	+	+	+	+	+
Were objective, standard criteria used for measurement of the condition?	+	+	+	+	-	+	+	-	+	-
Were confounding factors identified?	+	+	+	+	+	+	+	+	+	+
Were strategies to deal with confounding factors stated?	+	+	+	+	+	+	+	+	+	+
Were the outcomes measured in a valid and reliable way?	+	+	+	+	+	+	+	+	+	+
Was appropriate statistical analysis used?	+	+	+	+	+	+	+	+	+	+
Include	+	+	+	+	+	+	+	+	+	+

Some additional questions to assess the risk of bias

**Questions**	**Asakimori 2003**	**Beilby 2003**	**Calmarza 2015**	**Debette 2006**	**Djousse 2002**	**Doliner 2018**	**Fernandez-Miranda 2004**	**Hsieh 2008**	**Shin 2014**	**Slooter 2001**
Was recruitment conducted prospectively to avoid selection bias?	+	+	+	+	+	+	+	+	+	+
Were the study's population selection criteria sufficiently described?	+	+	+	+	+	+	+	+	+	+
Was the association between plaque and ApoE genes the primary outcome?	+	+	+	+	+	+	+	+	+	+
Were the imagers blinded to the clinical and genetic characteristics?	+	-	+	-	-	-	-	+	+	+
Did the authors explicitly clarify measurements for subjective terms	+	+	+	+	-	+	+	-	+	-

such as plaque thickness?										
Was plaque measured by >1 imager?	-	+	-	-	-	-	-	-	-	-
Was interrater reliability of plaque measurements assessed?	-	+	-	-	+	-	-	-	-	-
